# Clinical Profile and Factors Associated with Adverse Outcomes in Coronavirus Disease 2019-associated Mucormycosis: A Single-centre Study

**DOI:** 10.17925/EE.2023.19.2.2

**Published:** 2023-06-01

**Authors:** Krishna S Nair, Murali Alagesan, Dhanya Jose, Chidambaram Yoganathan, Rethinam Saravanan, Krishnasamy Karthikeyan, Karuppannasamy Divya, Dinesh Babu, Cyril Rajan, Joseph M Pappachan

**Affiliations:** 1. Department of General Medicine, PSG Institute of Medical Sciences and Research Center, Coimbatore, India; 2. Department of Community Medicine, Goa Medical College, Goa, India; 3. Department of Oral & Maxilofacial Surgery, PSGSSH, Coimbatore, India; 4. Department of ENT, PSG Institute of Medical Sciences and Research Center, Coimbatore, India; 5. Department of Opthalmology, PSG Institute of Medical Sciences and Research Center, Coimbatore, India; 6. Department of Dental Surgery, PSG Institute of Medical Sciences and Research Center, Coimbatore, India; 7. Department of Medicine & Endocrinology, Lancashire Teaching Hospitals NHS Foundation Trust, Preston, UK; 8. Faculty of Science, Manchester Metropolitan University, Manchester, UK

**Keywords:** Chronic kidney disease, coronavirus disease 2019, COVID-19-associated mucormycosis, haemoglobin A1C, intensive care unit, mucormycosis, rhino-orbito-cerebral mucormycosis, type 2 diabetes mellitus

## Abstract

**Background**: The coronavirus disease 2019 (COVID-19) pandemic was associated with an increased incidence of mucormycosis globally. However, the clinical pattern, epidemiologic features and risk factors for adverse outcomes are not well established. **Methods**: We performed a retrospective analysis of the data from patients hospitalized with proven mucormycosis between April 2021 and August 2021. Patients were managed with a multi-disciplinary approach involving medical, surgical, and comorbidity treatment. The clinical presentation, management details, complications and outcomes, including mortality, were reviewed from clinical records. **Results**: The mean age of presentation was 53.7 (± 11.8) years, and 88 (84.6%) were men. Of the 104 cases with COVID-19-associated mucormycosis, 97 (93.27%) patients had diabetes, and 80.8% had a haemoglobin A1C (HbA1c) of ≥6.4% at diagnosis. Seventy percent of diabetes cases experienced steroid-induced hyperglycaemia during treatment. Even with appropriate treatment, 17 (16.35%) patients died. High HbA1c and creatinine levels, presence of chronic kidney disease (CKD), need for intensive care unit admission, and orbital evisceration were the risk factors associated with high mortality on multivariate logistic regression analysis. Cox regression analysis revealed that the overall mortality increased by a factor of 12% with each 1 percentage point increase in HbA1c ≥6.4% (hazard ratio 1.12; 95% confidence interval 0.95– 1.31). The mortality risk was even higher when diabetes was associated with CKD (hazard ratio 1.82; 95% confidence interval 0.24–14.00). **Conclusion**: High HbA1c and creatinine levels, intensive care unit admission, CKD, and aggressive disease requiring orbital evisceration are the predictors of mortality in patients with COVID-19-associated mucormycosis. Patients with these risk factors should be managed more actively to reduce morbidity and mortality.

## Plan language summary

### Background

The global COVID-19 pandemic resulted in an increased incidence of infection with Black fungus (Mucormycosis). However, the disease characteristics, chances of spread of infection and complications are not well known.

### Aim of the study

We tried to find out the exact reasons behind this rise in the number of cases, whether anything particularly results in complications, and what can be done to avoid this.

### Methods

We collected the medical details and treatment outcomes of patients who were managed in our hospital by various medications and surgical operations from April 2021 to August 2021. We followed up these patients for 3 months after hospital admission and discharge to identify whether any factors particularly resulted in complications, including death.

### Findings

A total of 104 patients were treated for Black fungus infection during the period, of whom 88 were males. Ninety-seven patients had diabetes mellitus. Many patients who received steroid medications for the treatment of COVID-19 had worsening of their diabetes control. Even with appropriate treatment for COVID-19 and Black fungus infection, 17 patients died. Poor diabetes control, kidney problems, intensive care unit admission for aggressive treatment and surgical excision of eye(s) were found to increase the death risk. Diabetes with kidney failure was found to worsen the chance of death.

### Conclusion

COVID-19 patients with more severe disease, poor diabetes control, and kidney failure have higher chance of death from Black fungus infection, especially when their eyes are badly affected by the fungus.

Mucormycosis is an aggressive mycotic infection caused by a group of multinucleated, filamentous fungi belonging to the orders Mucorales and Entomophthorales.^1^ In humans, infection is usually caused by the inhalation or ingestion of sporangiospores or even by traumatic inoculation of conidia via puncture wounds or trauma.^1^ The disease usually occurs in immunocompromised hosts and can be associated with an aggressive course with angioinvasion and tissue destruction. The mortality associated with mucormycosis can be as high as 50% (or even more), especially when patients present with disseminated and highly invasive infections.^2^ The angioinvasive nature of the disease often results in thrombosis and vasculitis, causing necrosis of large areas of bones and tissues.^2^ Devitalizing of extensive areas of skin and soft tissues further worsens the prognosis, as drug penetration is poor in these tissues.^3,4^

Therefore, medical treatment coupled with extensive surgical debridement is often necessary for effectively managing these patients.^2,5^ Immunocompromised patients with uncontrolled diabetes mellitus, organ transplantation, chemotherapy, neutropenia, deferoxamine therapy, elevated serum iron levels, corticosteroid therapy and various haematological disorders are the most afflicted group.^3,6^

With the second wave of the coronavirus disease 2019 (COVID-19) pandemic, India faced a sudden rise in the incidence of mucormycosis.^7,8^ Most of these affected patients were those with active COVID-19 infections or those who acquired the fungal invasion during the recovery phase. This itself shows a significant correlation between these two infections.^9^ Due to this significant rise in cases during the COVID-19 pandemic, this condition was termed COVID-19-associated mucormycosis (CAM). Furthermore, the Government of India listed CAM under notifiable diseases, whilst most of the Indian state governments declared it an epidemic.^10^

Compared with other systemic fungal infections, mucormycosis is a relatively rare condition; therefore, it is difficult to acquire the sample size required for conducting randomized controlled trials to study the disease's epidemiological and prognostic characteristics effectively.^11^

We performed a retrospective descriptive clinical study to examine the disease characteristics and factors affecting adverse outcomes among patients treated for CAM in our hospital, and report the outcome data from this study.

## Materials and methods

**Figure 1:**
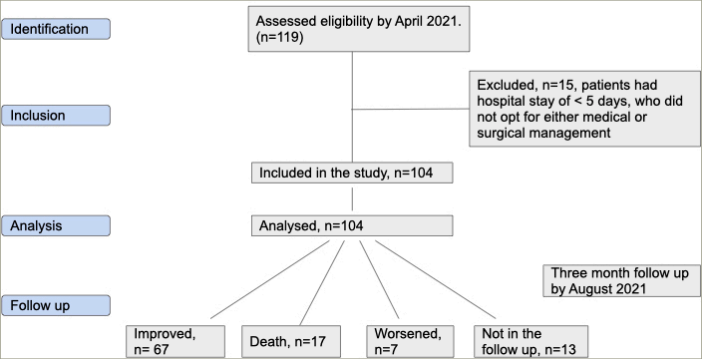
Strengthening the Reporting of Observational Studies in Epidemiology (STROBE) flow chart showing the detailed outline of the study

This retrospective descriptive clinical study was conducted among patients hospitalized with mucormycosis between April 2021 and August 2021 at the PSG Institute of Medical Sciences and Research Center located in Coimbatore, Tamil Nadu, India. The study was performed after obtaining formal institutional review board approval (reference number: PSG/ IHEC /2022/ APPR/EXP/041). The diagnosis of mucormycosis was based on clinical, radiological and/or biopsy samples of the infected tissue collected during the study period. All patients with a diagnosis were considered for inclusion in the study. Patients with a hospital stay of less than 5 days and those who opted for neither medical nor surgical management were excluded. *Figure 1* is a Strengthening the Reporting of Observational Studies in Epidemiology (STROBE) flow chart showing the detailed outline of the study.

A detailed history of symptoms was obtained from every participant's clinical chart. Clinical assessment data, including ear, nose, throat and cranial nerve examinations, and baseline haematological and biochemical investigations, were collected as part of the treatment. Every participant underwent contrast-enhanced magnetic resonance imaging of the brain and orbit, and computed tomography of paranasal sinuses to evaluate the severity of disease and tissue invasion, and these details were tabulated.

Details of the management of patients with mucormycosis are as follows. Each patient was managed with a multi-disciplinary approach, which comprised intravenous antifungal agents, surgical debridement when necessary, and treatment of underlying comorbid illnesses, such as diabetes mellitus and renal disease. A treatment protocol was formed locally in accordance with the Ministry of Health and Family Welfare of the Government of India.^10^ Conventional amphotericin B was the preferred antifungal agent at a dose of 1 mg/kg/day, owing to the very high price of the liposomal formulation in the region. For patients with renal impairment, dose adjustments were made according to the creatinine clearance (as calculated using the Cockroft-Gault equation). Patients who had moderate-to-severe renal failure (estimated glomerular filtration rate [GFR] 30–44 ml/min/1.73 m^2^), severe renal failure (GFR 15–29 ml/ min/1.73 m^2^) and renal failure (GFR <15ml/min/1.73 m^2^) were started on the liposomal formulation of amphotericin B if they were able to afford the high expense of the treatment. The administered dose of liposomal amphotericin B was 5 mg/kg/day; a dose of 10 mg/kg/day was prescribed in case of severe disease, such as rhino-orbito-cerebral mucormycosis (ROCM). Depending on the patient's response, the intravenous antifungal treatment was continued for between 3 and 4 weeks.

Patients were managed medically and surgically on a case-by-case basis. Additional surgical management, which included endoscopic sinus debridement, maxillectomy, orbital decompression, orbital evisceration or exenteration, and retro-orbital amphotericin injection, was implemented depending on the extent of involvement proved by radiological imaging and clinical response to disease control. All participants were monitored clinically and biochemically every 24-48 hours for treatment response and potential complications.

After 3-6 weeks, patients were switched over to oral antifungals – posaconazole or isavuconazole (according to patients’ financial affordability of the drug, isavuconazole being costlier) for 3 months. Patients who survived the acute illness were followed up over 3 months as outpatients following hospital discharge, with repeat imaging to assess the resolution of the disease.

The clinical and laboratory data were meticulously recorded for each case using a structured proforma. The data included demographic information, clinical symptoms and signs at first presentation, imaging details, surgical treatment administered, type of antifungal and its duration, follow-up imaging details, and morbidity and mortality data.

### Statistical analysis

To analyze the relationship between continuous variables, we made the initial approach of fitting logistic regression models with one covariate at a time and selecting the variables with the best fit for inclusion in the final regression analysis, along with other relevant covariates. We used advanced logistic regression to estimate the relationships between survival of patients with mucormycosis (the dependent variable) and other independent variables (i.e. age, sex, haemoglobin A1C [HbA1c] levels, chronic kidney disease [CKD], intensive care unit (ICU) admission, creatinine level at admission, presence of coronary artery disease and hypertension ). Any variable having a significant univariate test at some arbitrary level is selected as a candidate for the multivariate analysis. This statistical significance was based on the Wald test from logistic regression; a p-value cut-off point of 0.25 was selected, as more traditional cut-off points, such as p<0.05, can fail in identifying variables that are known to be necessary for our multivariate analysis (e.g. various comorbities, HbA1C levels, steroid use). Therefore, we selected all the variables whose p-value was <0.25, along with the variables of known clinical importance for the multinominal logistic regression analysis (e.g. uncontrolled diabetes, steroid use, ICU admission, presence of comorbidities).^12^

We assessed the probable association between the surgical procedure that patients underwent as part of the treatment of invasive mucormycosis during their hospital stay to examine if any particular treatment modality had an impact on overall survival. We also performed Cox regression analysis or proportional hazards regression, a statistical method used to model the relationship between survival time (or time to an event of interest) and one or more predictor variables. It is commonly used in medical and biological research to analyze the effects of various risk factors on time-to-event outcomes, such as the time until a patient dies or develops a particular health condition. The output of a Cox regression analysis typically includes estimates of the regression coefficients (β) for each predictor variable, as well as the hazard ratio (HR) and confidence interval (CI) associated with each coefficient. The HR indicates the relative increase or decrease in the risk of an event occurring associated with a one-unit increase in the predictor variable, while the CI provides a measure of the precision of the estimate.

Survival at 90 days was calculated from the date of diagnosis. Patients were followed up closely with enquiries over phone and frequent visits. We took an outcome analysis at the end of 90 days; if the patients were alive they underwent re-assessment, if they were lost to follow-up or had died they were categorised accordingly. Survival analysis for the continuous variable, HbA1c, was done. The survival curves were derived from Kaplan–Meier estimates. The Kaplan–Meier plots were produced in the style recommended by Morris et al. following the KMunicate study.^13^ They recommend to describe Kaplan–Meier curves with CIs to quantify uncertainty and also created an extended risk table (per treatment arm) depicting the number of study subjects at risk, events and censored observations over time.

**Table 1:** Demographic profile, disease severity and risk factors for rhino-orbito-cerebral mucormycosis

Parameter	Number (SD)
Age range	18–80 years
Mean age (years)	53.69 (11.83)
Male sex (%)	88.00 (84.62)
RT-PCR positivity at admission (%)	91.0 (87.5)
HRCT severity of COVID-19 (%)	
Mild Moderate Severe	34.00 (32.69)
	48.00 (46.15)
	22.00 (21.15)
Oxygen requirement during hospitalization (%)	52 (50)
ICU admission for COVID-19 disease (%)	27.00 (25.96)
Steroid administration (%)	73.00 (70.19)
Diabetes mellitus (%)	97.00 (93.27)
Hypertension (%)	30.00 (28.85)
Coronary artery disease (%)	15.00 (28.85)
Chronic kidney disease (%)	3.00 (2.89)
Cerebrovascular accident (%)	2.00 (1.92)

*Chronic kidney disease is defined as kidney damage or as estimated glomerular filtration rate of less than 60 ml/min/1.73 m^2^ persisting for 3 months or more, irrespective of the cause.*

*COVID-19 = coronavirus disease 2019; HRCT = high resolution computed tomography; ICU = intensive care unit; RT-PCR = reverse transcription-polymerase chain reaction; SD = standard deviation.*

The statistical analyses were conducted using the R software package and jamovi software for statistical computing. Statistical significance was set at p<0.05; all tests were two sided.

## Results

During the study period, 104 cases of mucormycosis were identified, 88 of whom were men (the demographic and clinical data of the cases are detailed in *Table 1*). The age range was 18–80 years, and the mean (standard deviation) age was 53.69 (± 11.83) years. Of the participants, 91 tested positive for severe acute respiratory syndrome coronavirus 2 by reverse transcription-polymerase chain reaction at the time of admission, indicating active COVID-19 infection. The remainder of the cases with previous positive tests by reverse transcription-polymerase chain reaction for COVID-19 were referred from other regional hospitals. Eighty-three patients had histopathological confirmation of mucormycosis from the biopsy of infected tissue samples, and the remainder had clinical and radiological evidence for the diagnosis (staining and detection of *Mucor* in necrotic tissues can often be difficult and may explain negative histology in these cases).

Seventy-three patients (70.19%) with moderate-to-severe disease were treated with steroids per the Indian Ministry of Health and Family Welfare guidelines for the management of COVID-19 infection.^10^ Seventeen patients (16.35%) died even with appropriate treatment. The baseline clinical and laboratory profiles are shown in *Table 1*. Of the 97 (93.27%) patients with type 2 diabetes mellitus (T2DM), 80.8% had HbA1c levels ≥6.4% at the time of mucormycosis occurrence; furthermore, 11.6% were newly diagnosed with diabetes during hospitalization. Steroid-induced hyperglycaemia (SIH) was common: 70% of patients with T2DM experienced SIH, thus requiring increased doses of antidiabetic medication or the addition of new medications for the control of hyperglycaemia.

**Table 2:** Staging and clinical presentation of coronavrus disease 2019-related rhino-orbito-cerebral mucormycosis

Parameter	Number (%)		
**ROCM staging**			
I. Involvement of nasal mucosa	22 (21.15)		
II. Paranasal sinuses involved			
Frontal	98 (94.23)		
Maxillary	87 (83.65)		
Ethmoid	76 (73.08)		
Sphenoid	83 (79.81)		
III. Involvement of the orbit			
Right orbit	32 (30.77)		
Left orbit	35 (33.65)		
Bilateral	9 (8.65)		
IV. CNS involvement	25 (25.04)		
**Ocular signs and symptoms related to mucormycosis at presentation**	Right	Left	Bilateral
	Number (%)	Number (%)	Number (%)
Ptosis	26 (25)	20 (19.23)	3 (2.88)
Conjunctival congestion	21 (20.19)	18 (17.31)	4 (3.85)
Conjunctival chemosis/ edema	15 (14.42)	12 (11.54)	1 (0.96)
Proptosis	20 (19.23)	11 (10.58)	1 (0.96)
Diminution of vision	27 (25.96)	23 (22.12)	14 (13.46)
Ophthalmoplegia	26 (25)	14 (13.46)	4 (3.85)
Fundus abnormality	7 (6.73)	3 (2.88)	2 (1.92)
Oedema of eyelids	32 (30.77)	22 (21.15)	6 (5.77)
**Nasal signs and symptoms at presentation**			
Bloody nasal discharge	22 (21.15)		
**Facial signs and symptoms at presentation**			
Facial pain	75 (72.12)		
Facial swelling	58 (55.77)		
Facial discoloration	67 (64.42)		
**Involvement of oral cavity at presentation**			
Palate involvement	40 (38.46)		
**CNS signs and symptoms at presentation**			
Altered sensorium	4 (3.85)		
Hemiplegia	0 (0)		
Headache	74 (71.15)		
**Other symptoms**			
Fever	14 (13.46)		
Cough	10 (9.62)		
Dyspnoea	11 (10.58)		

*CNS = central nervous system; ROCM = rhino-orbito-cerebral mucormycosis.*

Staging of ROCM^14^ and its clinical features are presented in *Table 2*.

Histopathological diagnosis of the cases indicated invasive mucormycosis (shown by tissue and vascular invasion) in 75 cases (72.11%), and 6 cases (5.77%) also showed coinfection with aspergillosis. Surgical treatment was administered according to the severity of tissue invasion. Details of disease characteristics, treatment provided, and treatment outcomes are shown in *Table 3*.

**Table 3:** Histopathology, management and outcomes of mucormycosis

Parameter	Number (%)
**Histopathological and/or microbiological diagnosis**	83 (79.81)
Invasive mucormycosis	75 (72.11)
Invasive mucormycosis + aspergillosis	6 (5.77)
Aspergilloma	2 (1.92)
No microbiological diagnosis	21 (20.19)
**Anti-fungal treatment**	
Retroorbital amphotericin B	19 (18.27)
IV conventional amphotericin B	89 (85.57)
IV liposomal amphotericin B	15 (14.42)
Posaconazole	64 (61.54)
Isavuconazole	30 (28.85)
Voriconazole	2 (1.92)
**Type of surgery for the treatment of mucormycosis**	
FESS / FESS + orbital decompression / FESS + medial maxillectomy	102 (98.08)
1. FESS	54 (51.92)
2. FESS + orbital decompression	22 (21.15)
Maxillectomy	39 (37.50)
Orbital evisceration	2 (1.92)
Orbital exenteration	8 (7.69)
**Outcome**	
Improved	67 (64.42)
Deceased	17 (16.35)
Not on follow-up	13 (12.50)
Worsened	7 (6.73)

*FESS = Functional endoscopic sinus surgery; IV = intravenous.*

Patients who survived the acute illness were followed up for 3 months after hospital discharge and were reassessed clinically and radiologically.

The logistic regression analysis revealed that a high HbA1c level at admission had a statistically significant negative association with overall survival (odds ratio [OR] 0.79; p=0.047). ICU admission had a statistically significant negative association with overall survival (OR 0.09; p<0.001). Similarly, high serum creatinine levels at admission was also negatively associated with survival (OR 0.47; p=0.039). None of the other risk factors had a statistically significant association with overall survival at 3 months (*Table 4*).

Furthermore, we assessed the probable association between the surgical procedure that patients underwent as part of the treatment of invasive mucormycosis during their hospital stay to examine if any particular treatment modality had an impact on overall survival (*Table 5*). None of the surgical procedures, other than orbital evisceration (OR 2.49E+7; p<0.001), appeared to be negatively correlated with survival.

Cox regression analysis revealed that higher HbA1c levels were associated with a reduced probability of survival. The overall mortality increased by a factor of 12% with each 1 percentage point increase in the HbA1c above 6.4% (HR 1.12; 95% CI 0.95–1.31). Mortality risk was found to be even higher among patients with diabetes when CKD was also present (HR 1.82; 95% CI 0.24–14.00)

**Table 4:** Results of the multinominal logistic regression analysis

Predictor	Estimate	SE	Z-value	p-value	Odds ratio
Intercept	8.9516	2.9734	3.0106	0.003	7720.50
Male sex	-0.799	0.9678	-0.8256	0.409	0.45
Higher HbA1c	-0.2415	0.1214	-1.9885	0.047*	0.79
Higher age	-0.0462	0.0381	-1.2151	0.224	0.95
ICU admission	-2.3917	0.7208	-3.3183	<0.001***	0.09
Presence of CAD	-0.2282	0.9428	-0.242	0.809	0.80
Presence of hypertension	-0.4213	0.792	-0.532	0.595	0.66
Presence of CKD	2.3942	2.6927	0.8892	0.374	10.96
High creatinine on admission (serum creatinine >1.2)	-0.763	0.3695	-2.0646	0.039*	0.47

The multinominal logistic regression analysis showing the causal relationship between the outcome and risk factors. Negative coefficients suggest that as the independent variable increases, the dependent variable tends to decrease. Note: Signif. codes: 0 ' *** ' 0.001 ' ** ' 0.01 ' * ' 0.05 '.' 0.1 ' ' 1

*CAD = coronary artery disease; CKD = chronic kidney disease; HbA1c = haemoglobin A1C; ICU = intensive care unit; SE = standard error.*

**Figure 2:**
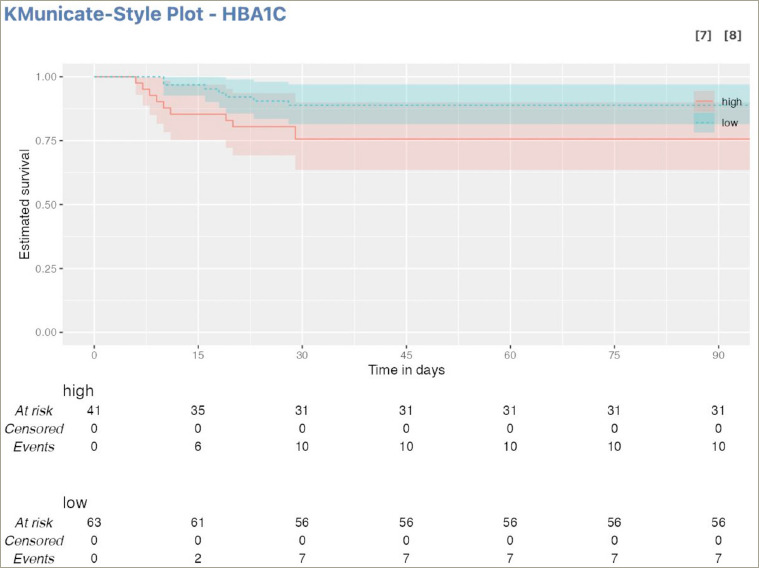
Kaplan–Meier unicate plot showing the relationship between haemoglobin A1C level and survival of the patients

The Kaplan-Meier curve was constructed by first dividing the study population into subgroups with high HbA1C and low HbA1C. Then, for each subgroup, the proportion of individuals who had survived up to a given time point was plotted on the y-axis, while the corresponding time points were plotted on the x-axis. An extended risk table (per treatment arm) depicting the number of study subjects at risk, events and censored observations over time was also created. It revealed that high baseline HbA1c (HbA1c >6.4%) was associated with low estimated survival over time, while low baseline HbA1c (HbA1c ≤6.4%) showed a higher estimated survival over time (*Figure 2*).

**Table 5:** Result of the logistic regression analysis

Predictor	Estimate	SE	Z-value	p-value	Odds ratio
Intercept	-1.622	1.183	-1.371	0.17	0.19749
Orbital evisceration	17.03	1.102	15.451	<0.001***	2.49E+7*
Orbital exenteration	-1.238	1.405	-0.881	0.378	0.28985
Maxillectomy	-0.352	0.876	-0.401	0.688	0.70359
FESS with orbital decompression	-0.151	1.216	-0.124	0.901	0.85996
FESS with medial maxillectomy	-0.718	1.243	-0.578	0.563	0.48752
FESS	-1.828	1.089	-1.679	0.093	0.16076

*The logistic regression analysis showing the causal relationship between the survival and treatment parameters.*

*2.49E+7=2.49 x 10^7^

†Note: Signif. codes: 0 ' *** ' 0.001 ' ** ' 0.01 ' * ' 0.05 '.' 0.1 ' ' 1

*FESS = functional endoscopic sinus surgery; SE = standard error.*

*HbA1c = haemoglobin A1C.*

Although we analyzed the available data of our patients who were followed up for 90 days after the diagnosis of mucormycosis, no change in survival was observed after 30 days (prior to which all deaths occurred).

## Discussion

This article reported the data from one of the largest single-centre studies on CAM with a robust assessment of clinical-pathological correlations, management aspects, and factors related to adverse clinical outcomes. In this study, significant predictors of mortality in patients with CAM were high HbA1c and creatinine levels, the presence of CKD, and the requirement for ICU admission for aggressive management. Orbital evisceration was also associated with significant mortality risk, probably indicating the severity of their illness.

Patients in our cohort may have been predisposed to developing CAM by preexisting diabetes mellitus or prediabetic state, steroid therapy, and compromised immune functions due to COVID-19, as observed in other studies.^7,9,15^ Corticosteroid use is particularly implicated in the aetiopathogenesis of CAM, as a systemic immune response to invasive fungal infections, such as mucormycosis and aspergillosis, are found in a very high proportion of patients.^14^ Steroid therapy may exacerbate gross alterations in lymphocyte and macrophage functions in COVID-19, and make patients particularly vulnerable to these kinds of invasive fungal diseases.

Data from our study concerning mean age at presentation (54 years) and male preponderance (88%) reflect the data from a larger study (53 years and 74%, respectively) recently conducted in India by Muthu et al. on 1,733 patients.^9^ The reason for male preponderance in our study is not clear; however, smoking, which is more prevalent among men,^16^ increases fungal invasion and thus may be a risk factor.^17^ Although 93.27% of our patients had diabetes or a history of diabetes, only 80.8% had an HbA1c level of ≥6.4%; this value is very similar to the prevalence of diabetes among patients with CAM reported by Muthu et al. (80.9%),^9^ and by Singla et al. (78.9%),^18^ another single-centre study from north India. However, we observed a significantly lower mortality rate (16.4%) compared with these two studies (28.6 and 33.9%, respectively).^9,18^ Better survival rates in our cohort. Wide variations in the morbidity and mortality risk among patients with CAM were also observed by other studies from different regions of the world.^19–21^

ICU admission was identified as a risk factor for lower survival rate in our study, indicating that the severity of COVID-19, as such, could have impacted the mortality risk in various studies reported worldwide. Indiscrete and prolonged use of corticosteroids among patients with COVID-19 were identified as important risk factors for CAM and CAM-related death rates.^9,22,23^ The widespread use of dexamethasone therapy for hypoxic COVID-19, as demonstrated by the results of the RECOVERY trial, may have led to a surge in the indiscriminate use of corticosteroids for patients with even mild disease. This could potentially explain the wide discrepancy in morbidity and mortality statistics for CAM.^24^

As observed in other studies,^9,22,23^ we also found uncontrolled hyperglycaemia in diabetes mellitus to be a significant predictor of mortality in our cohort. Similarly to other studies,^25,26^ our research found that patients with ROCM and diabetes had worse mortality risk when CKD or acute kidney injury coexisted. Although increasing age was a predictor of excess mortality risk in the study by Muthu et al.,^9^ we did not observe such an association in logistic regression analysis in our cohort, presumably because of the smaller sample size. However, we found that a better disease outcome was associated with aggressive surgical debridement of ROCM, as has been observed by others.^9,27,28^

We acknowledge some limitations of the present study that may affect the generalizability of our findings to day-to-day clinical practice. First, given the significant association between COVID-19 and a high risk of ROCM, it is important to note that the sample size in our study is relatively small. Second, the epidemiology of the COVID-19 pandemic has varied widely due to the presence of different strains. This may have affected its incidence, and markedly changed the clinical picture of its complications, including mucormycosis, after the second wave of the pandemic in 2021. Moreover, the risk factors and disease manifestations of COVID-19 vary significantly across different regions of the world and population subgroups;^29^ therefore, the presentation of ROCM and its complications may have also been different at different times and in different regions. On-going surveillance of case management from different regions of the world may provide us with more insight into these issues.

Since our study was conducted in a setting where complete data on patients was well maintained, the risk of bias in our retrospective study is not much higher than in a prospective cohort study. By themselves, Kaplan–Meier curves are not an efficient way to account for confounders in retrospective studies. Therefore, we also performed the Cox proportional hazards regression analysis appropriate for the censored survival data, to account for the measured potential confounders. Despite these limitations, our data provide broad prognostic and management perspectives to clinicians treating CAM and ROCM worldwide, highlighting the importance of our study.

## Conclusions

CAM is a serious consequence of the COVID-19 pandemic and can be associated with a high risk of morbidity and mortality. Diabetes mellitus, renal impairment, and the need for ICU admission for treatment are associated with a higher risk of mortality. Early diagnosis, prompt administration of medical therapy, and appropriate surgical debridement of invasive ROCM may improve the excess mortality risks associated with CAM. Future large-scale studies are required to provide us with better insight into managing this potentially lethal COVID-19-associated disease.
